# Generating Real‐World Evidence From AI Deployed in Health Systems

**DOI:** 10.1002/lrh2.70131

**Published:** 2026-11-04

**Authors:** Lily Poursoltan, Aaron Boussina, Jie Cao, Eileen Kim, Poorva Satish Bedmutha, Hetvi Majmudar, Mattheus Ramsis, Alexander Khalessi, Karandeep Singh

**Affiliations:** ^1^ Rady School of Management University of California San Diego California USA; ^2^ Joan and Irwin Jacobs Center for Health Innovation UC San Diego Health San Diego California USA; ^3^ Division of Biomedical Informatics, Department of Medicine University of California San Diego California USA; ^4^ Division of General Internal Medicine, Department of Medicine University of California San Diego California USA; ^5^ Department of Electrical and Computer Engineering University of California San Diego California USA; ^6^ Department of Biology University of California San Diego California USA; ^7^ Division of Cardiovascular Medicine, Department of Medicine University of California San Diego California USA; ^8^ Department of Neurological Surgery University of California San Diego California USA

**Keywords:** artificial intelligence, generative AI, implementation, learning health system, predictive AI, real world evidence

## Abstract

**Background:**

Health systems increasingly deploy predictive and generative artificial intelligence (AI) to improve care delivery and outcomes. Predictive AI models estimate the risk of future events to inform targeted interventions, while generative AI systems extract, summarize, and communicate information and may automate selected tasks. Routine deployment generates clinical, operational, and interaction data that can support evaluation of the effectiveness and safety of AI‐enabled workflows.

**Methods:**

We review approaches to generating real‐world evidence (RWE) from routine deployment of predictive and generative AI across four implementation configurations: silent deployment, human‐in‐the‐loop (HITL), human‐on‐the‐loop (HOTL), and automated. For each configuration, we describe the evaluation questions, routinely generated data, and feasible study designs.

**Results:**

Each implementation configuration supports different forms of evidence about model performance, workflow effectiveness, human oversight, and safety. For both predictive and generative AI, silent deployment supports assessment of local performance and subgroup differences. Once predictive AI drives action, evaluating the effectiveness of an implemented workflow (HITL, HOTL, or automated) generally requires causal inference approaches. For generative AI, HITL implementations can generate data on edits, overrides, time on task, usability, cognitive burden, and safety‐relevant failure modes. HOTL and automated generative AI implementations can generate data on escalation performance, human follow‐up work, downstream outcomes, and safety signals.

**Conclusion:**

AI implementation should be paired with methods for generating RWE on the effectiveness and safety of AI‐enabled workflows. In HOTL and automated configurations, escalation performance and residual human workload become particularly important components of evaluation.

## 1 Introduction

Learning health systems increasingly deploy artificial intelligence (AI) within routine care delivery. Because these tools are digitally embedded in clinical workflows, their use generates clinical, operational, and interaction data that can support evaluation. In this Brief Report, we use real‐world evidence (RWE) to refer to evidence generated by analyzing data arising from the routine deployment and use of AI‐enabled workflows. We focus on research questions concerning the safety and effectiveness of these workflows and local quality‐improvement decisions about refinement. Regulatory, accountability, and high‐stakes reliability claims may require additional standardized measurement, independent adjudication, or purpose‐designed data collection beyond the scope of this framework [1, 2].

Persistent resource constraints motivate the use of AI in health care. Clinical demand exceeds available capacity, requiring continual reallocation of human effort across patient populations and tasks [1, 2, 3, 4]. AI is incorporated into care delivery workflows in two broad forms: predictive and generative AI. Predictive AI uses clinical and operational data to estimate the risk of future events, with interventions prioritized for high‐risk patients. Generative AI extracts, summarizes, and communicates information and can be connected to external knowledge sources and software tools to support multistep or agentic workflows. Such workflows may automate selected tasks while escalating cases that meet predefined criteria for clinical review [3, 4]. The need for automation is underscored by a recent Utah program allowing AI models to refill prescription medications [5] and the nascent ARPA‐H Agentic AI‐EnableD CardioVascular CAre TransfOrmation (ADVOCATE) program [6]. Across these forms of implementation, a central question is how learning health systems should generate rigorous RWE on AI‐enabled workflows with different forms of human oversight and automation.

Several reporting guidelines address health AI evaluation, primarily for predictive AI, including model development and validation during silent deployment (i.e., TRIPOD+AI, PROBAST+AI), early‐stage clinical evaluation and usability (i.e., DECIDE‐AI), and randomized trials of AI‐enabled interventions (SPIRIT‐AI, CONSORT‐AI) [7, 8, 9, 10, 11]. These frameworks are helpful for evaluating AI but do not specify how to generate RWE from routine deployment outside trial contexts, where selective intervention, feedback loops, and user adaptation may alter the data‐generating process [3, 8, 9, 10, 11, 12]. In such settings, commonly tracked performance measures can change for reasons unrelated to model drift, complicating effectiveness assessment and post‐deployment monitoring [12]. Evaluation strategies must therefore distinguish performance in silent settings from effectiveness in implemented workflows, where confounding and feedback loops may affect observed performance if the causal relationships among AI outputs, interventions, and outcomes are not considered [12]. Generative AI also yields informative interaction traces, including what clinicians edit, accept, and reject and what actions follow. These traces enable direct measurement of workflow consequences that are difficult to infer from benchmarks alone [13, 14, 15].

In this Brief Report, we describe approaches to generating RWE from AI deployed in learning health systems, organized by how AI outputs are used and how human oversight is structured. For predictive and generative AI, we map key evaluation questions, routinely generated data, and feasible study designs across four implementation configurations: silent deployment, human‐in‐the‐loop (HITL), human‐on‐the‐loop (HOTL; asynchronous human oversight), and automated. These configurations are not necessarily sequential, and greater automation is not inherently the preferred endpoint. For many applications, HITL may remain the appropriate long‐term configuration.

## 2 AI Implementation Configurations

We use the following operational definitions, which distinguish configurations according to how model outputs affect clinical workflows and how human oversight is structured.

### 2.1 Silent Deployment

The AI tool runs prospectively in the background while its outputs are logged but not shown to decision‐makers or used to guide care. Evidence is generated by comparing model outputs with observed outcomes or other task‐specific reference standards.

### 2.2 Human‐in‐the‐Loop

A human reviews AI outputs (predictive or generative) and determines whether and how to use them. Evidence is generated from review behavior, including edits, overrides, acceptance or rejection, explicit ratings or annotations, downstream actions, and associated workload or quality indicators. In this framework, “human” refers to health system staff rather than patients. Patients nevertheless remain important stakeholders in the design and evaluation of health system AI.

### 2.3 Human‐on‐the‐Loop

The AI tool initiates an action without prior human review, while health system personnel provide asynchronous oversight. Cases may be escalated based on predefined uncertainty or exception criteria, and selected actions may be reviewed shortly after they occur. Evidence is generated from system outputs and actions, escalation decisions, findings from human review, and failure modes identified through audit and adjudication.

### 2.4 Automated

The AI tool performs tasks at scale using established escalation pathways. The criteria for escalation should be established before automated implementation, potentially through prior HOTL evaluation. Evaluation focuses on downstream outcomes, residual human workload, and ongoing safety monitoring.

Across these configurations, evidence selection should be anchored to three questions: (a) what claim or decision the evidence is intended to support, such as a research or local quality‐improvement claim or a decision about continuation, refinement, scaling, automation, or decommissioning; (b) what measures can be captured in routine systems and are relevant to that purpose; and (c) which study designs support interpretable inferences given the causal structure of the workflow, including feedback loops. For local quality improvement, measures should align with the goals of the intervention and include outcome, process, and balancing measures. RWE may be generated through prospective or retrospective analyses of data arising from routine care. The appropriate approach depends on the intended inference, data completeness, and potential sources of bias. These considerations are described in Figure 1.

**FIGURE 1:**
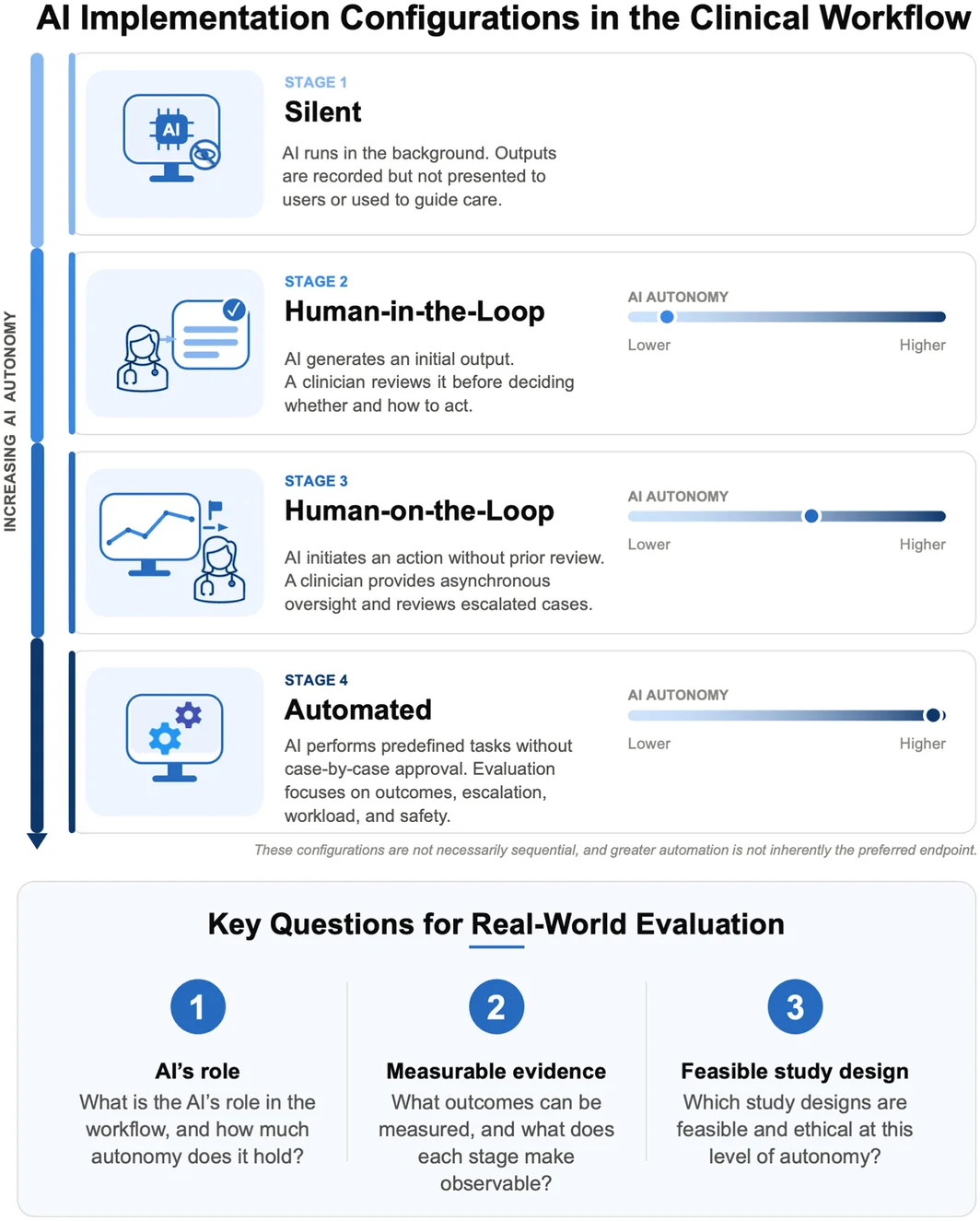
AI implementation configurations in clinical workflows. These configurations are not necessarily sequential. The implementation configuration influences which measures are available in routine data and which study designs are feasible for generating real‐world evidence.

## 3 Generating RWE for Predictive AI

### 3.1 Silent Deployment

Silent deployment allows assessment of local validity and subgroup performance before model outputs are incorporated into a clinical workflow. For example, local validation of a silently deployed predictive model can determine whether performance is sufficient for clinical use and estimate expected alert frequency, an important consideration when assessing alert burden [16]. Core evidence includes discrimination, calibration, decision‐analytic measures such as net benefit, threshold‐dependent measures such as alert frequency, sensitivity, specificity, positive and negative predictive values, and subgroup analyses.

### 3.2 Human‐in‐the‐Loop Implementation

When predictive models trigger outreach, triage, alerts, or other actions, they become part of an intervention. In this configuration, the key question is whether the AI‐enabled workflow improves outcomes. Having a HITL means that risk scores produced by the model are reviewed by a human before being acted upon. High concordance between risk estimates and user actions may reflect appropriate reliance, but it may also reflect overreliance.

Determining whether an AI‐enabled workflow is effective requires causal evaluation, ideally through randomization when feasible and, in routine deployment, often through quasi‐experimental causal inference designs. HITL workflows can induce feedback loops, in which the intervention triggered by the AI model alters observed model performance [12, 17]. Changes in observed performance may therefore reflect the intervention rather than algorithmic drift. Algorithmic drift refers broadly to changes in model performance over time as the underlying data or relationships between predictors and outcomes change and is an important target of post‐deployment monitoring [17, 18]. For example, if an AI‐triggered intervention prevents adverse outcomes among patients identified as high risk, the observed association between predictions and outcomes may weaken even when the workflow is beneficial [12, 17]. Observed changes may therefore reflect intervention effects, workflow adaptation, changes in case mix, or algorithmic drift [17, 18]. Monitoring and RWE generation overlap in both the data they use and the questions they address, particularly in HOTL and automated implementations. Routine monitoring data may contribute to RWE when analyzed to address a defined clinical, operational, or implementation question using methods appropriate to the intended inference.

Causal methods can help distinguish the effect of the intervention from other sources of change. Randomization is preferred when ethically, logistically, and financially feasible. When not possible, quasi‐experimental observational designs provide another option. Examples of such designs include multivariable regression in the context of a staggered implementation [19], Bayesian structural time‐series analyses [20], and regression discontinuity [21]. These examples illustrate that evaluating effectiveness is distinct from model validation and is rarely accomplished through silent deployment alone. Purpose‐designed qualitative or mixed‐methods data collection, including interviews, surveys, or workflow observation, can complement RWE by clarifying adoption, user reliance, and workarounds that shape observed effects and post‐deployment monitoring signals [22].

### 3.3 Human‐on‐the‐Loop Implementation

When predictive AI is used in a HOTL approach, the model drives an action that is subsequently (i.e., asynchronously) reviewed by a human. Certain models, such as conformal prediction models, may produce an “I don't know” when the training data are not sufficiently representative for a given prediction [23]. Compared with HITL, evaluation of HOTL predictive AI places greater emphasis on whether cases are appropriately escalated for human review and whether errors remain detectable and correctable when identified during asynchronous oversight.

### 3.4 Automated Implementation

Automated implementations can be evaluated using methods similar to those used for HITL and HOTL implementations, with additional emphasis on the consequences of false‐positive and false‐negative predictions and the extent of escalation to human review.

## 4 Generating RWE for Generative AI

### 4.1 Silent Deployment

In clinician‐facing deployments where generative systems produce content for review, such as summaries, extracted clinical elements, or drafted text for documentation and communication, RWE can be generated directly from analysis of the content before it is shown to humans. Common evaluation methods include comparison with a reference standard, structured rubric‐based review conducted manually or supplemented by an LLM‐as‐judge approach, and assessment of whether clinically important statements are present or omitted. In this configuration, AI‐drafted content can be reviewed for accuracy, appropriateness, and alignment with its intended use.

### 4.2 Human‐in‐the‐Loop Implementation

Once AI‐drafted content is shown to humans for review, RWE can be generated directly from observable review behavior. The extent and pattern of clinician modifications can indicate where outputs require clarification, contextualization, or correction. When paired with telemetry on review and edit time, these patterns can be used to estimate workload, including effort attributable to verification and revision. This evidence supports targeted improvement by identifying high‐effort tasks and recurrent content failure modes that warrant prompt optimization, model refinement, interface redesign, or selective constraints on use [13, 14].

One challenge in using humans as the reference standard is that reviewers may make errors or overrely on AI‐generated content. Minimal editing may therefore reflect either model accuracy or insufficient scrutiny. In a study of generative AI models abstracting hospital quality measures [24], nearly half of the discrepancies between AI outputs and human abstractors were attributable to human error, with the AI producing the correct result. RWE is strengthened when discrepancies are adjudicated to distinguish AI error from human error.

### 4.3 Human‐on‐the‐Loop Implementation

Patient‐facing conversational AI systems exemplify settings in which direct review of every output before delivery may not be feasible because a clinical team member is not present in each conversation. In these settings, voice recordings, transcripts, and evaluations of system responses can provide RWE on performance and safety. Assessing whether the system appropriately identifies cases requiring human escalation is particularly important for determining whether the current oversight configuration is safe and whether escalation criteria require refinement.

### 4.4 Automated Implementation

Automation does not imply that all work is delegated to an AI system. In an automated implementation of generative AI, the system performs initial work, such as chart review for billing or routine calls to patients, and uses established escalation criteria to identify cases requiring human follow‐up. Human work remains necessary for oversight and resolution of escalated cases.

Once escalation pathways have demonstrated sufficient reliability, automated deployments should quantify residual human follow‐up work, assess downstream clinical and operational outcomes, and maintain ongoing safety monitoring. Illustrative examples of AI‐enabled workflows, routinely generated data, corresponding evaluation questions, and potential learning health system actions across the four implementation configurations are summarized in Table 1.

**TABLE 1:** Illustrative evaluation of predictive and generative AI across implementation configurations.

AI type	Implementation configuration	Illustrative use case	Routinely generated data and measures	Primary evaluation question	Potential learning health system action
Predictive	Silent implementation	An electronic health record‐based clinical deterioration or sepsis risk model operating without displaying risk estimates to clinicians	Model predictions; observed outcomes; discrimination and calibration; threshold‐specific performance; performance across prespecified subgroups; expected alert volume	Does the model demonstrate adequate local performance at clinically relevant thresholds, with acceptable alert volume and no clinically important differences across relevant subgroups?	Recalibrate the model; revise the alert threshold or target population; conduct additional local validation; or defer implementation
Predictive	Human‐in‐the‐loop	A deterioration‐risk alert reviewed by a clinician before outreach, diagnostic evaluation, or treatment	Risk estimates; alert delivery and acknowledgment; clinician response; time to action; intervention delivered; clinician workload; patient outcomes	Does the AI‐enabled workflow change clinician behavior and improve clinical or operational outcomes compared with usual care?	Refine the alert, interface, threshold, or response protocol; strengthen implementation support; scale, constrain, or discontinue the workflow
Predictive	Human‐on‐the‐loop	A model‐triggered workflow initiates protocolized outreach or triage, with flagged or sampled cases reviewed asynchronously by clinical staff	Actions initiated; escalation decisions; findings from human review; corrective actions; time to correction; downstream outcomes	Are cases requiring human review escalated appropriately, and are errors detected while corrective action remains possible?	Revise escalation criteria; increase audit frequency; restrict the range of model‐triggered actions; or strengthen correction pathways
Predictive	Automated	A model‐triggered workflow initiates a predefined, low‐risk clinical or operational action under established oversight and escalation procedures	Actions initiated; consequences of false‐positive and false‐negative predictions; escalation frequency; downstream outcomes; safety events; residual human workload	Does the automated workflow remain effective and safe, and are the consequences of incorrect predictions acceptable?	Continue or scale the workflow; narrow its scope; modify oversight; temporarily suspend use; or decommission the system
Generative	Silent implementation	Draft replies to patient messages, clinical summaries, or abstracted quality‐measure elements generated but not shown to users	Generated content; reference assessments; task‐specific rubric scores; factual errors; omissions; unsupported statements; performance across prespecified subgroups	Is the generated content sufficiently accurate, complete, and appropriate for its intended use before it is introduced into the workflow?	Modify the prompt, model, knowledge source, or task definition; narrow the intended use; or defer implementation
Generative	Human‐in‐the‐loop	AI‐generated draft replies to patient messages or draft clinical documentation reviewed by a clinician before use	Acceptance or rejection; edits and edit type; review time; overrides; usability measures; downstream actions; safety‐relevant errors	How does the system affect review workload, and does clinician‐approved content remain accurate, appropriate, and safe?	Refine the prompt, model, interface, or information sources; identify tasks requiring greater scrutiny; or restrict use in higher‐risk contexts
Generative	Human‐on‐the‐loop	A patient‐facing conversational system conducts routine outreach or addresses common questions, with selected conversations escalated for asynchronous review	Audio recordings or transcripts; system responses and actions; escalation triggers; missed or delayed escalations; human follow‐up; patient disposition; safety events	Do escalation criteria reliably identify conversations requiring clinical review, and is asynchronous oversight sufficient to identify and address failures?	Revise escalation logic; narrow the conversational scope; increase human oversight; improve follow‐up workflows; or retain the current configuration
Generative	Automated	An AI system performs routine chart abstraction, administrative review, or patient outreach with established escalation pathways	Task completion; unresolved cases; escalation frequency; human follow‐up time; correction rates; downstream clinical or operational outcomes; safety signals	Does the automated workflow complete the intended task accurately and efficiently, with acceptable escalation performance, safety, and residual human workload?	Scale the workflow; modify escalation pathways or staffing; constrain the task scope; pause deployment; or decommission the system

*Note:* Examples are illustrative rather than prescriptive. The configurations are not necessarily sequential, and greater automation is not inherently the preferred endpoint. Routinely generated data become RWE when analyzed using methods appropriate to the intended inference, clinical and operational context, and causal structure of the AI‐enabled workflow.

## 5 Conclusion

Learning health systems need RWE strategies that match how AI is implemented and overseen. Silent deployment is important for establishing local validity and identifying subgroup differences, but it cannot determine whether AI‐enabled workflows improve outcomes once predictions or generated content drive action. Once AI outputs influence care, generating credible RWE requires study designs that support causal inference, together with evaluation of escalation pathways and measurement of human workload, including residual follow‐up work. Organizing RWE generation by implementation configuration supports rapid learning cycles and informs whether an AI‐enabled workflow should be continued, refined, scaled, automated, or decommissioned.

## Conflicts of Interest

The authors declare no conflicts of interest.

## Data Availability

Data sharing not applicable to this article as no datasets were generated or analyzed during the current study.
